# Goethite Hinders Azo Dye Bioreduction by Blocking Terminal Reductive Sites on the Outer Membrane of *Shewanella decolorationis* S12

**DOI:** 10.3389/fmicb.2019.01452

**Published:** 2019-06-25

**Authors:** Gang Zhao, Enze Li, Jianjun Li, Feifei Liu, Fei Liu, Meiying Xu

**Affiliations:** ^1^Guangdong Provincial Key Laboratory of Microbial Culture Collection and Application, Guangdong Institute of Microbiology, Guangdong Academy of Sciences, Guangzhou, China; ^2^State Key Laboratory of Applied Microbiology Southern China, Guangzhou, China

**Keywords:** goethite, azo dye, bioreduction, mineral interface, *Shewanella decolorationis* S12

## Abstract

Iron (hydr)oxides are the most ubiquitous Fe(III)-containing minerals in the near-surface environments and can regulate organic pollutant biotransformation by participating in bacterial extracellular electron transfer under anaerobic conditions. Mechanisms described so far are based on their redox properties in bacterial extracellular respiration. Here, we find that goethite, a typical iron (hydr)oxide, inhibits the bioreduction of different polar azo dyes by *Shewanella decolorationis* S12 not through electron competition, but by the contact of its surface Fe(III) with the bacterial outer surface. Through the combined results of attenuated total reflectance (ATR) Fourier transform infrared spectroscopy, two-dimensional correlation spectroscopy, and confocal laser scanning microscope, we found that the outer membrane proteins MtrC and OmcA of strain S12 are key binding sites for goethite surface. Meanwhile, they were identified as the important reductive terminals for azo dyes. These results suggest that goethite may block the terminal reductive sites of azo dyes on the bacterial outer membrane to inhibit their bioreduction. This discovered role of goethite in bioreduction provides new insight into the microbial transformation processes of organic pollutants in iron (hydr)oxide-containing environments.

## Introduction

Under anaerobic conditions, various organic pollutants such as azo dyes and halogenated hydrocarbons can be reduced by acting as electron acceptors in bacterial anaerobic respiration (Schwarz et al., 2012; Venkidusamy et al., 2016; Xu et al., 2019). This biotransformation process plays an important role in biogeochemical cycling and environmental bioremediation (Flynn et al., 2014; Venkidusamy and Megharaj, 2016; Sheu et al., 2018), and is affected by the insoluble iron (hydr)oxides, which are abundant in soil and sediments (Cornell and Schwertmann, 2003). The insoluble iron (hydr)oxides (e.g., goethite and magnetite) are widely recognized as extracellular electron transfer conduits in the microbial reductive transformation of organic pollutants because their surface Fe(III) can accept electrons from bacterial cells (Borch et al., 2005; Aulenta et al., 2013; Wang et al., 2018). They may either inhibit the transformation by directly competing for electrons with organic electron acceptors (Yager et al., 1997), or boost it by becoming biogenic Fe(II), which can mediate electron transfer from bacterial cells to organic compounds (Amonette et al., 2000; Cao et al., 2012). Besides, biogenic Fe(II) is a source of hydroxide radical (⋅OH), which attacks organic pollutants indiscriminately (Chen et al., 2016; Yan et al., 2016).

In addition to accepting electrons, Fe(III) on the surface of iron (hydr)oxide is a binding site for bacterial cells. It can specifically bind to bacterial outer membrane components such as proteins and phosphate groups (Parikh and Chorover, 2006; Wei et al., 2016). In organisms capable of extracellular electron transport such as *Shewanella* and *Geobacter*, the hemeproteins on the bacterial outer membrane had shown high affinity to iron (hydr)oxide surface (Xiong et al., 2006; Sheng et al., 2016). They are also the terminal proteins of the electron transport chain that transports electrons from the cell to the extracellular electron receptors such as soluble Fe ions and high polar organics (Ross et al., 2009; Liu et al., 2017). Furthermore, increasing evidence showed that the terminal reductive proteins on the bacterial outer membrane transfer electrons to Fe(III) on the surface of insoluble iron (hydr)oxide much more slowly than to the soluble electron acceptors. For example, the reduction rates of soluble Fe ions by purified terminal reductases and bacterial cells (*Shewanella oneidensis* MR-1) were up to 10^5^–10^6^ and 10^3^–10^4^ times more than that of goethite (Ross et al., 2009). For *Shewanella decolorationis* strain S12, no Fe(OH)_3_ reduction was detected after 7 days incubation, but its efficiency in azo dye decolorization reached 97.6% after 7 h (Xu et al., 2007). It remains unknown whether the contact of the hemeproteins with Fe(III) on the surface of iron (hydr)oxide will affect the electron transfer from these terminal reductive proteins to organic compounds. In natural environments, iron (hydr)oxide nanoparticle aggregates are often observed on the surfaces of bacterial cells (Fortin et al., 1998; Luef et al., 2013). The effects of this behavior on extracellular electron transfer are essential for understanding microbial reduction of organic pollutants in iron-rich environments.

Thus, this study aims to explore the influence of the interactions between the surface of iron (hydr)oxide particles and bacterial cells on the bioreduction of azo dyes. Methyl orange and methyl red, which differ in one sulfonic acid substituent, were chosen and the former could be reduced only extracellularly while the latter could cross the cytoplasmic membrane and be reduced inside or outside cells (Liu et al., 2015, 2017). We measured the bioreduction kinetics of azo dyes in systems with *S. decolorationis* S12 as the functional bacteria and goethite as model iron (hydr)oxide. We hypothesized that goethite, rather than serving as a terminal extracellular electron acceptor, blocks the terminal reductive proteins on the outer surface of strain S12 via direct contact, and that the bioreduction rate or extent of methyl orange might be more susceptible to the presence of goethite. To examine the role of Fe(III) on goethite surface in the bioreduction process, we masked it with a phosphate group. For the molecular-scale mechanisms between bacterial outer membrane and goethite, we explored their interfacial interactions by using *in situ* attenuated total reflectance (ATR) Fourier transform infrared (FTIR) spectroscopy, two-dimensional correlation spectroscopy (2D-COS), and confocal laser scanning microscope (CLSM).

## Materials and Methods

### Chemicals and Mineral

Methyl red, methyl orange, and 4-(2-hydroxyethyl) piperazine-1-ethanesulfonic acid (HEPES) were purchased from Sigma–Aldrich. Goethite was synthesized by the procedure of Atkinson et al. (1967). Phosphate-adsorbed goethite was prepared by mixing goethite (final concentration of 1000 mg L^–1^) with a KH_2_PO_4_ (100 mg L^–1^) for 48 h and stored before use.

The *n*-octanol-water partition coefficient (log *K*_*OW*_) is an important parameter of hydrophobicity, which can be used to assess the distribution trends of organic pollutants in both polar and nonpolar phases (Liu et al., 2015). Log *K*_*OW*_ of methyl red and methyl orange were obtained from Estimation Program Interface (EPI) Suite (USEPA, United States Environmental Protection Agency, Washington, 2017).

### Bacteria and Growth Conditions

*Shewanella decolorationis* strain S12T (CCTCCM203093T = IAM 15094^T^) and its mutant strains (△*mtrC* and △*omcA*) were preserved in our laboratory. All strains were grown aerobically in sterilized LB medium at 28°C. Then the bacterial suspensions were centrifuged at 6000 × *g* for 10 min at 28°C and washed three times with 30 mM HEPES buffer. The harvested cells were resuspended in HEPES buffer for subsequent use.

### Bioreduction Assays

Experiments were conducted in 8 ml serum bottles capped with Teflon-coated rubber stoppers. Experimental preparations and measurements were performed in an anoxic chamber (Electrotek AEP) filled with a gas mix (N_2_:H_2_ 90:10). Each bottle was filled with 5 ml HEPES buffer containing various combinations of strain S12 (about 10^7^ cells ml^–1^), goethite (0–1000 mg L^–1^), methyl red (1 mM), or methyl orange (1 mM), and was deoxygenated by bubbling with N_2_ for 30 min. Sodium formate was the electron donor, whose concentration (10 mM) was high enough to support regular bioreduction of both dyes. At predetermined time intervals (0.5, 2, 4, 6, 8, 10, 12, and 14 h), the bottles were taken out and analyzed for the concentration of methyl red or methyl orange. The concentrations of methyl orange and methyl red were determined using a UV–vis spectrophotometer by recording the absorption at 460 and 430 nm (Chen et al., 2010; Liu et al., 2017). Control reactors were prepared as described above but without bacterial inoculants. In addition, strain S12 mutants △*mtrC* and △*omcA* were used to reduce azo dye to assess the role of related proteins. All treatments were conducted in triplicates.

Competition for electrons between goethite and azo dyes was evaluated in reactors in the presence of strain S12, sodium formate, goethite, and either azo dye. As the colorimetric assay of Fe(II) can be disturbed by azo dyes, the incubation was extended to 14 h to reduce the interference. The 0.5 N HCl-extractable Fe(II) was analyzed using the ferrozine assay.

To characterize the effect of goethite surface Fe(III) on bioreduction of azo dyes, the reductive kinetics of two dyes was also evaluated in the presence of phosphate-adsorbed goethite, and data were collected and analyzed in the same manner as previously stated. Phosphate has been shown to effectively weaken the interaction strength between iron (hydr)oxides and bacterial cells (Appenzeller et al., 2002). The phosphate group can bind Fe(III) at the surface, displacing coordinated hydroxyl group and/or water molecules (Tejedortejedor and Anderson, 1990; Elzinga and Sparks, 2007), and thus reduce contact probability of Fe(III) with the bacterial outer membrane (Supplementary Figures S2, S3). The phosphate adsorption amount of goethite surface was about 8.09–39.46 mg g^–1^ (Torrent et al., 1990), and thus excessive phosphate (100 mg L^–1^) by reference was added. We measured the phosphate concentration of the supernatant of phosphate in phosphate-adsorbed goethite system after strain S12 spiked (about 10^7^ cells ml^–1^), and there was no significant difference compared with bacteria-free bacteria treatment (*p* > 0.05).

### ATR-FTIR Experiments and 2D-COS Analysis

Attenuated total reflectance Fourier transform infrared measurements were performed with a horizontal ATR cell (Pike Technologies, Inc.), equipped with a ZnSe crystal element, in a Bruker Tensor II FTIR spectrometer. All spectra were averaged with 256 scans at 4 cm^–1^ resolution.

The spectra of bacterial cells were collected by subtracting the spectrum of background solution. A relatively high concentration of bacterial cells (∼10^8^ cells ml^–1^) was used to clearly show FTIR signals of the bacterial cell. Background solution was passed through the flow cell at the rate of 2 ml min^–1^ until the equilibrium was established and the spectrum was used as background spectrum. Then S12 cells were spiked and constantly stirred with a magnetic stirrer. The interfacial spectra of S12 cells were measured with goethite film on the surface of ZnSe crystal. The film was prepared by drying 1 ml of a 1 g L^–1^ dispersed goethite suspension at 60°C for 6 h under an N_2_-atmosphere in a similar way as the previous report (Elzinga et al., 2012; Elzinga and Kretzschmar, 2013; Wei et al., 2016). The coated film was pre-equilibrated with background solution at a flow rate of 2 ml min^–1^ until there was no significant difference in the spectra. A background spectrum containing the absorbance of the solution, goethite, and ZnSe crystal was then obtained. Collection of interfacial spectra started as bacterial suspension entered the flow cell, for up to 4 h.

For the S12-goethite system, IR spectra collected with an increment of 20 min were analyzed using 2Dshige software version 1.3 (Kwansei Gakuin University, Japan). Synchronous and asynchronous maps in 2D-COS were created and the origin of band pair (υ_1_, υ_2_) can be known from the sign of synchronous and asynchronous correlation peak [Φ(υ_1_,υ_2_) and Ψ(υ_1_,υ_2_)] according to Noda’s rules (Noda and Ozaki, 2005). Briefly, if synchronous and asynchronous correlation peaks have the same signs, the spectral change at υ_1_ precedes that at υ_2_. As for the opposite sign, the order is reversed.

### Confocal Laser Scanning Microscope (CLSM) and Transmission Electron Microscopy (TEM)

To determine the role of the terminal reductive proteins MtrC and OmcA in bacterial outer membrane–goethite interactions, we used CLSM to observe the behavior of S12 and its mutants on the surface of goethite through a parallel plate flow chamber. Goethite particles were attached to microscope glass slides (25 mm wide, 75 mm long, and 1 mm thick) and the coating method was similar to ATR-FTIR experiment. The glass slide was loaded onto the parallel plate flow chamber as a cover. A peristaltic pump was used to connect the flow chamber and circulate deoxygenated HEPES buffer containing sodium formate (10 mM) and bacterial cells (about 10^7^ cells ml^–1^) at a rate of 2 ml min^–1^ for 4 h. Then flow was switched to HEPES buffer for 5 min and the glass slide was removed and washed by HEPES to eliminate non-adhering bacteria cells. The slide was treated with LIVE/DEAD BacLight staining kit and imaged by CLSM (LSM 700, Zeiss). Twenty-four 319.53 μm^2^ images were collected from three replicates. The number of adhering bacterial cells was analyzed using an image analysis software (Image J, NIH).

For TEM observation, bacterial cells were incubated with 100 mg L^–1^ goethite for 4 h and then washed three times in HEPES buffer, fixed in 2% glutaraldehyde, dried in ethanol, and embedded in epoxy resin. Thin sections were cut using an ultramicrotome. Then samples were imaged on a Hitachi H-7650 with an accelerating voltage of 80 kV.

## Results

### Effects of Goethite on Bioreduction of Azo Dyes

The molecular structure of methyl orange and methyl red differs in a high polar sulfonic acid group. Log *K*_*OW*_ of methyl orange and methyl red were −0.66 and 3.83, respectively. Based on the basic principle of “like dissolves like,” the difference of log *K*_*OW*_ demonstrated the predominantly high polarity and hydrophilic nature of methyl orange and the low polarity of methyl red.

**FIGURE 1:**
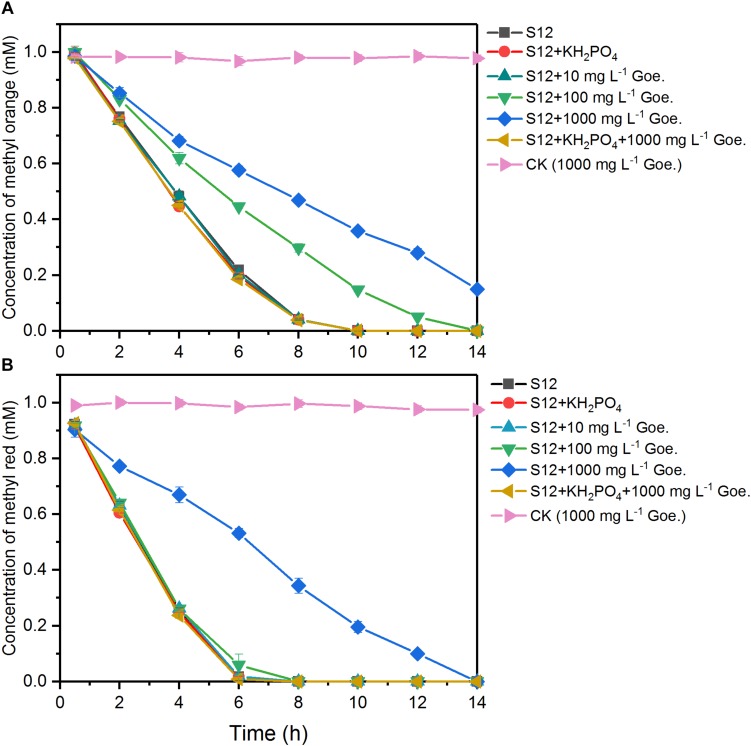
Biological reduction of methyl orange **(A)** and methyl red **(B)** by strain S12. The controls (CK) were prepared under the same conditions but without strain S12 inoculation. The plots are the average of triplicate samples and error bars indicate the standard deviation.

As shown in Figure 1, 1 mM methyl orange and methyl red could be completely decolorized by strain S12 within 10 and 6 h, respectively, while no decolorization was detected in the abiotic reactors, suggesting that the azo dye reduction resulted from the biotic process. The first-order rate constant of methyl orange bioreduction (0.28 h^–1^) was lower than that of methyl red (0.39 h^–1^), indicating that the high polar sulfonic acid group impeded the bioreduction.

We tested the azo dye reduction abilities of mutant strains lacking MtrC or OmcA, which are the terminal reductive proteins on the outer membrane for the reduction of extracellular soluble iron (Ross et al., 2009). In the absence of MtrC or OmcA, 75 and 23% of methyl orange remained unreduced over 10 h (Supplementary Figure S1) and the ΔmtrC and ΔomcA complemented strains had a recovery of azo dye-reducing ability (data not shown). These results suggest that MtrC and OmcA are responsible for the reduction of methyl orange. It also indicates that methyl orange with a high polarity sulfonic acid group was mainly reduced extracellularly. In contrast, only 36 and 22% of methyl red remained after 6 h of cultivation of the MtrC- and OmcA-lack mutant strains, suggesting that the reduction of methyl red was less affected by the knock-out of MtrC.

The bioreduction of methyl orange and methyl red at different concentrations of goethite (0–1000 mg L^–1^) is shown in Figure 1. No significant impacts were observed in the system with 10 mg L^–1^ goethite. For methyl red, goethite significantly suppressed the bioreduction rate only at a concentration of 1000 mg L^–1^. For methyl orange, the first-order reduction rate constant was 0.28 h^–1^ in the absence of goethite and decreased to 0.18 and 0.11 h^–1^ in the presence of 100 and 1000 mg L^–1^ goethite. These results indicated that the inhibition of azo-reduction was proportional to the concentration of goethite added, and higher susceptibility was observed in the reduction of high polarity methyl orange.

### Effects of Goethite-Surface Fe(III) on Bioreduction of Azo Dyes

To examine whether Fe(III) on goethite surface would compete with azo dye for electrons, we measured the levels of Fe(II) in various systems. Since the given electron donor (sodium formate) did not reduce Fe(III), Fe(II) in the system was solely attributed to biogenic production by strain S12. After 14 h of incubation, the total concentration of Fe(II) was 0.054 ± 0.006 and 0.077 ± 0.008 mM, respectively, in the 1000 mg L^–1^ goethite-S12 systems with methyl orange or methyl red. Note that each molecule of sodium formate provides two electrons when oxidized (Hong et al., 2007) which is equivalent to two Fe(II) ions getting reduced. The electrons captured by Fe(III) accounted for merely 0.27 and 0.39% of the total electrons calculated by the consumption of sodium formate, respectively. These results strongly suggested that the reducing capacity of strain S12 for Fe(III) on goethite surface was weak under our experimental conditions.

We also tested the effect of goethite-surface Fe(III) on microbial azo dye reduction by masking the goethite surface with phosphate group binding. No significant differences were observed in the reduction rates between treatments with and without phosphate (Figure 1), suggesting that phosphate did not alter the electron flow to azo dyes. In the following assay, we replaced the 1000 mg L^–1^ goethite with phosphate-masked goethite (1000 mg L^–1^) and the inhibition of goethite on methyl orange and methyl red reduction disappeared. The first-order reduction rate constants of methyl orange and methyl red were 0.28 and 0.39 h^–1^, respectively, showing no difference from the goethite-free treatments. Together, these results suggested that goethite-surface Fe(III) inhibited the bioreduction of azo dyes by the contact with bacterial cells, rather than serving as a competitive electron acceptor.

### The Contact of Strain S12 With Goethite Surface

**FIGURE 2:**
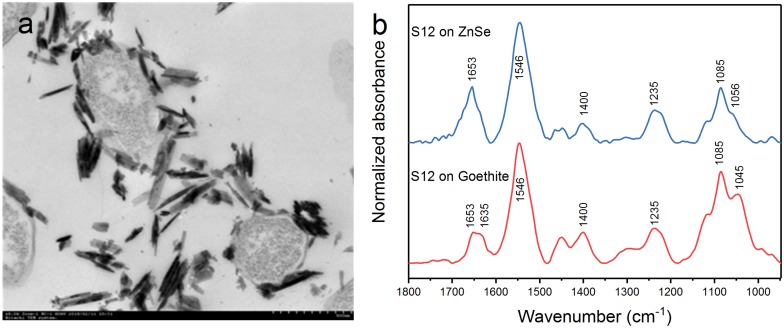
TEM of S12-goethite aggregations when strain S12 and goethite were pre-equilibrated for 4 h **(a)**; ATR-FTIR spectra of S12 cells on ZnSe and goethite after 4 h equilibrium **(b)**.

Transmission electron microscopy showed that the outer membrane of bacterial cells was coated with massive goethite particles (Figure 2a). *In situ* ATR-FTIR spectroscopy was further used to explore strain S12 interfacial behaviors on the goethite surface. As shown in Figure 2b, the ATR-FTIR spectrum of goethite-free S12 cells was similar to previous reports in the frequency range of 1800–950 cm^–1^ (Jiang et al., 2004; Elzinga et al., 2012). The bands between 1800 and 1200 cm^–1^ contain the amide groups [amide I (1653) and amide II (1546)] and carboxyl [e.g., deprotonated carboxyl groups (1400), carboxyl groups (1235)] (Jiang et al., 2004). The bands within ∼1150 and 950 cm^–1^ are a complex composite of phosphate region, like υ(C-O-P, P-O-P) and υ_s_(${\mathrm{PO}}_{2}^{-}$) (Parikh and Chorover, 2006). Compared to the case with ZnSe only, distinct changes were observed in the range of phosphate groups in the spectra of strain S12 on goethite surface and a prominent band at 1045 cm^–1^ was found (Figure 2b). This band was attributed to the stretching vibrations of P-OFe in ${\mathrm{Fe}}_{2}\,{\mathrm{PO}}_{4}^{-x}$ (Tejedortejedor and Anderson, 1990), which indicated that P-moieties of bacterial cells were involved in the interactions between strain S12 and Fe(III) at goethite surface through the information of inner-sphere complexes.

**FIGURE 3:**
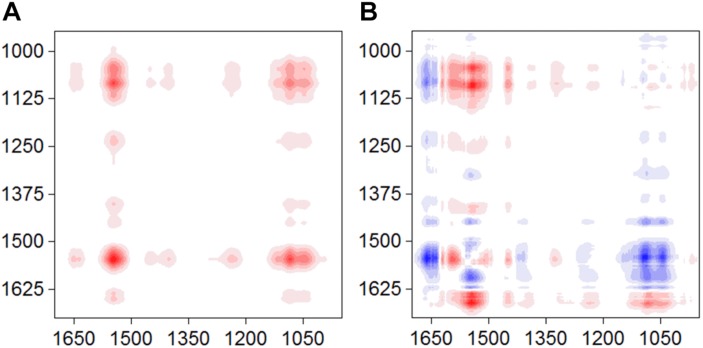
Synchronous **(A)** and asynchronous **(B)** contour maps obtained from the time-dependent ATR-FTIR spectra of strain S12 attached on goethite for 4 h equilibration.

The synchronous and asynchronous maps for the S12-goethite system were shown in Figure 3. We chose the signal of amide II (1546) as a characteristic signal for bacterial surface proteins because the amide I band was often perturbed by water-related adsorptions (Shephard et al., 2010). In the synchronous map, all auto-peaks were positive (Figure 3A) at 1546/1045 cm^–1^, indicating same sign of variation in peak intensity of amide II and υ(P-OFe). In the asynchronous map, the positive cross peak at 1546/1045 cm^–1^ was found (Figure 3B). According to Noda’ rule, the sequence of the spectral change in intensity of amide II occurred before υ(P-OFe). Our result suggested that, when bacteria cells were introduced to the surface of goethite, the proteins at the outer cell surface firstly recognized or approached the interface. Then, phosphate group on bacterial envelope formed the inner-sphere surface complexes on goethite.

**FIGURE 4:**
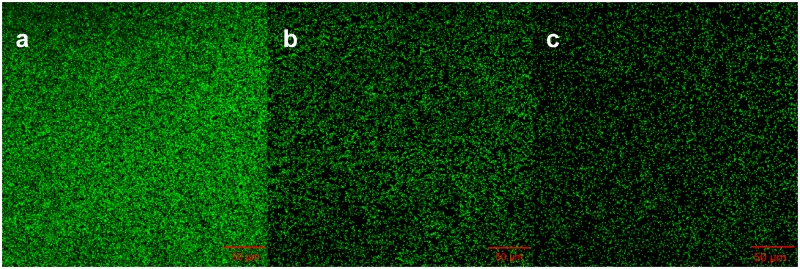
Representative CLSM images of strain S12 **(a)**, S12 (△*mtrC*) **(b)**, and S12 (△*omcA*) **(c)** on goethite-coated glass slides after 4 h. The area of each image is 319.53 μm^2^. The surface cell density of strain S12, △*mtrC*, and △*omcA* was 0.176 ± 0.018, 0.077 ± 0.006, and 0.06 ± 0.005 cells μm^–2^, respectively.

**FIGURE 5:**
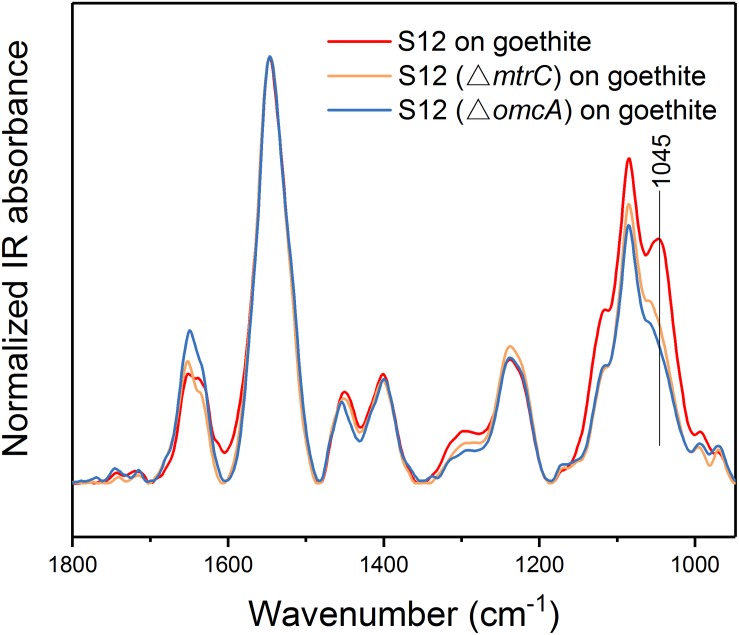
ATR-FTIR spectra of strain S12, S12 (△*mtrC*), and S12 (△*omcA*) on goethite.

To understand whether proteins at the outer cell surface like MtrC and OmcA were in contact with Fe(III) on goethite surface, we collected CLSM images and the ATR-FTIR spectra of S12 mutants (△*mtrC* and △*omcA*) on goethite (Figures 4, 5). The surface cell density of △*mtrC* (0.077 ± 0.006 cells μm^–2^) and △*omcA* (0.06 ± 0.005 cells μm^–2^) on goethite-coated glass slides was significantly lower than strain S12 (0.176 ± 0.018 cells μm^–2^) (*p* < 0.001), suggesting MtrC and OmcA are key binding sites for goethite surface. In addition, different from the ATR-FTIR spectrum of S12 wild-type on goethite, υ(P-OFe) at 1045 cm^–1^ was not observed in the spectra of △*mtrC* or △*omcA* on goethite. It meant that, when MtrC and OmcA were not present on the S12 outer membrane, Fe(III) on goethite surface was not in contacted with phosphate moieties of the bacterial outer membrane to form a covalent bond. Furthermore, the number of bacteria on the mineral surface greatly reduced when surface Fe(III) was masked by phosphate, indicating that goethite-surface Fe(III) was the binding site of bacteria cell (Supplementary Figure S3). These results strongly suggested that MtrC and OmcA could participate directly in the interaction between the bacterial out membrane and Fe(III) on goethite surface by co-bounding to goethite surface.

## Discussion

This study presents evidence that goethite does not act as a competitive electron acceptor to hinder azo dye bioreduction and suggests a novel mechanism that the contact of insoluble iron (hydr)oxide particles with bacterial outer membrane can affect the bioreduction process. We found that the goethite–bacterial interaction was the driving factor that limits the reduction rates of methyl orange and methyl red. ATR-FTIR, 2D-COS, and CLSM analyses suggested that the terminal reductive sites (e.g., MtrC and OmcA) for azo dye on the outer membrane were directly involved in the interaction between goethite-surface Fe(III) and the outer surface of strain S12. These findings allow us to reassess the previously proposed mechanisms for the role of insoluble iron (hydr)oxides in the bioreduction of organic compounds.

The presence of insoluble iron (hydr)oxides could significantly inhibit the bioreduction of organic compounds (Roden, 2003; Zhang and Weber, 2009). A widely accepted explanation is that insoluble iron (hydr)oxides act as an electron sink for extracellular electron transfer and thus compete with organics for electrons. It was documented that the iron (hydr)oxides-reduction ability of *Shewanella* species was weak when no additional extracellular electron shuttles (e.g., riboflavin and flavin mononucleotide) were added (von Canstein et al., 2008). Little Fe(II) was generated in our bioreduction experiment where no electron shuttle was added. Electron competition was largely disabled but still, a significant inhibition was observed. These results support that goethite hinders azo dye bioreduction not by electron competition.

Our results showed that the contact of goethite-surface Fe(III) with the bacterial outer membrane was the critical factor for inhibiting azo-reduction and higher susceptibility was observed in the reduction of high polarity methyl orange. We identified two terminal reductive proteins MtrC and OmcA on the outer membrane of strain S12, and the knock-out of either protein would result in a significant drop in the reduction rate of methyl orange. Previous studies have shown that MtrC and OmcA were the main components of electron transport chain in *Shewanella*, and were responsible for soluble iron reduction and current production in microbial fuel cells (Ross et al., 2007; Hartshorne et al., 2009; Ross et al., 2009). The interaction between goethite-surface Fe(III) and bacterial outer membrane likely reduced the number of electrons transferred from the terminal reductive sites to methyl orange.

**FIGURE 6:**
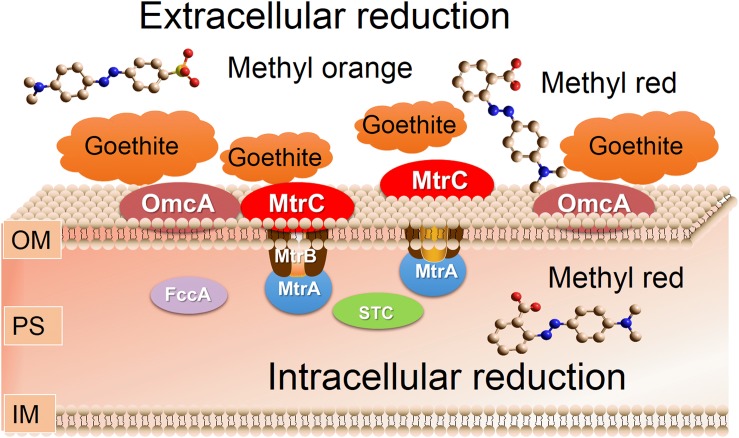
Schematic representation of the proposed effect mechanisms of goethite on bioreduction of methyl orange and methyl red by *S. decolorationis* S12.

Furthermore, our results suggested that MtrC and OmcA were also key binding sites for the attachment of bacteria to the goethite surface. This was consistent with previous studies showing that MtrC and OmcA had high affinity to insoluble iron (hydr)oxides (Xiong et al., 2006; Sheng et al., 2016). Lower et al. (2008) reported that the binding motif (Ser/Thr-Pro-Ser/Thr) near the terminal heme-binding domain of both MtrC and OmcA might form hydrogen bonding with the hydroxylated hematite surface to facilitate their adsorption processes. However, little Fe(II) being found during bioreduction of azo dyes meant that little electrons were directly transferred from MtrC or OmcA to goethite surface Fe(III). Ross et al. (2009) found that MtrC and OmcA were not responsible for the reduction of insoluble iron (hydr)oxides via direct contact, and the reduction rate of soluble Fe ions by purified MtrC or OmcA is up to 10^5^–10^6^ orders than that of goethite. Thus, the decreased amounts of electron from MtrC or OmcA to methyl orange may be due to the blockage of goethite-surface Fe(III) to the terminal reductive sites on the outer membrane of strain S12 (Figure 6). This mechanism can also explain differences in the bioreduction rate of methyl orange and methyl red in the presence of high concentration goethite. The reduction of methyl orange by strain S12 was more susceptible to high concentration goethite than the reduction of methyl red was, because the blockage happened to only the outer membrane and methyl orange was obligatory for extracellular reduction.

## Conclusion

Our study suggests that goethite may block the key sites where strain S12 transfers electrons to extracellular environment. This is in contrast to the widely accepted concept insoluble iron (hydr)oxides affect the microbial reduction of organic substrates as electron transfer conduits. It is known that microorganisms often be associated with iron (hydr)oxides in soil and sediment environment (Ferris et al., 1986; Grantham et al., 1997; Luef et al., 2013). Thus, the ubiquitous reducers of organic pollutants would encounter a similar situation and the interaction of bacterial outer membrane with iron (hydr)oxides might influence dramatically on the biogeochemical cycling. More importantly, extra attention should be paid to the blocking effect in the practical applications of microbial reduction technologies which add iron (hydr)oxides or under iron-rich environments.

## Data Availability

All datasets generated for this study are included in the manuscript and/or the Supplementary Files.

## Author Contributions

GZ and MX designed the study and wrote the paper. GZ and EL operated the experiments. JL, FFL, and FL discussed the results. All authors agreed to be accountable for the content of the work.

## Conflict of Interest Statement

The authors declare that the research was conducted in the absence of any commercial or financial relationships that could be construed as a potential conflict of interest.
