# Supplementary material for: Goethite Hinders Azo Dye Bioreduction by Blocking Terminal Reductive Sites on the Outer Membrane of Shewanella decolorationis S12
Source: Front Microbiol. 2019 Jun 25;10:1452. doi: 10.3389/fmicb.2019.01452 (PMC6604703; doi:10.3389/fmicb.2019.01452)
Supplement: Supplementary file 1 [file Data_Sheet_1.docx]

Supplementary Material

Goethite hinders azo dye bioreduction by blocking terminal reductive sites on the outer membrane of *Shewanella decolorationis* S12

Gang Zhao^1, 2^, Enze Li^1, 2^, Jianjun Li^1, 2^, Feifei Liu^1, 2^, Fei Liu^1, 2^, Meiying Xu^1, 2*^

^1^ Guangdong Provincial Key Laboratory of Microbial Culture Collection and Application, Guangdong Institute of Microbiology, Guangzhou 510070, China

^2^ State Key Laboratory of Applied Microbiology Southern China, Guangzhou 510070, China

*** Correspondence:**Meiying Xu
[xumy@gdim.cn](mailto:xumy@gdim.cn)

This file contains 4 pages, including 3 figures.





**Fig. S1 Biological reduction of methyl orange (a) and methyl red (b) by S12, S12 (*△mtrC*) and S12 (*△omcA*).** The controls (CK) were prepared under the same conditions but without S12 inoculation. The plots are based on the average data of triplicate samples and error bars indicate standard deviation of the replicates.





**Fig. S2 ATR-FTIR spectra of phosphonate solution on ZnSe and goethite after 4 h equilibrium.** ATR-FTIR data of phosphonate on ZnSe and goethite after 4 h equilibrium were collected and analyzed in the frequency range of 1200-900 cm^-1^, which contained the signal of stretching bands of orthophosphate adsorption complexes at iron (hydr)oxide surfaces. The absorption bands of phosphonate complexes on goethite at 1025, 1008, 948 showed some differences from the spectra of phosphonate solution, and the bands at 1025 and 1008 cm^-1^ could be assigned to the vibrations of P-O in Fe_2_PO_4_ and P-OFe in Fe_2_HPO_4_ (Tejedortejedor M.I., Anderson M.A. The protonation of phosphate on the surface of goethite as studied by CIR-FTIR and electrophoretic mobility. *Langmuir* **1990**, *6*, 602-611; Arai Y., Sparks D.L. ATR–FTIR spectroscopic investigation on phosphate adsorption mechanisms at the ferrihydrite–water interface. *J. Colloid. Interf. Sci.* **2001**, *241*, 317-326). This indicated conformational changes of the phosphate group on the cytoplasmic membrane after being adsorbed to goethite, which can be explained by the formation of inner-sphere complexes with Fe(III) at goethite surface.


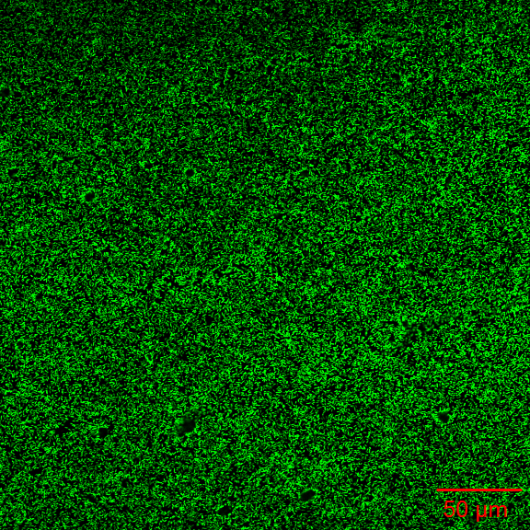

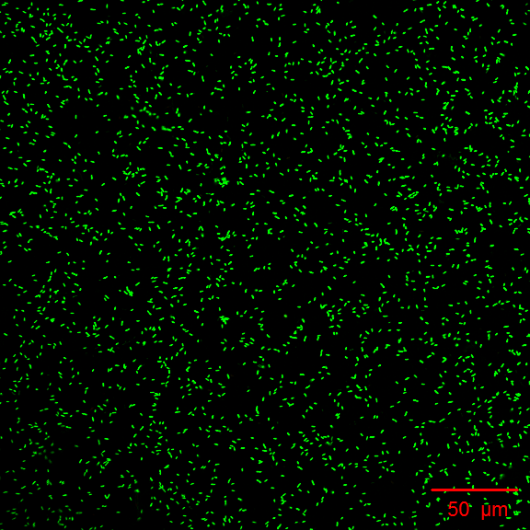


**a**

**b**

**Fig. S3 Representative epifluorescence microscopy images of strain S12 attached on goethite (a) and phosphate-adsorbed goethite (b).** The number of bacterial cells on goethite and on phosphate-adsorbed goethite were 0.176±0.018 and 0.027±0.005 cells μm^-2^, respectively. This result indicated phosphate could effectively reduce the number of bacterial cells being attached on goethite surface.
